# A cluster randomized trial of an organizational process improvement intervention for improving the assessment and case planning of offenders: a Study Protocol

**DOI:** 10.1186/2194-7899-2-1

**Published:** 2014-01-08

**Authors:** Michael S Shafer, Michael Prendergast, Gerald Melnick, Lynda A Stein, Wayne N Welsh, the CJDATS Assessment Workgroup

**Affiliations:** Arizona State University, School of Social Work, 500 N. Third Street, Suite 200, Phoenix, AZ 85004-2135 USA

**Keywords:** Correctional treatment systems, Assessment, Case planning, Change teams, Facilitators, Multi-site cluster randomized design

## Abstract

**Background:**

The Organizational Process Improvement Intervention (OPII), conducted by the NIDA-funded Criminal Justice Drug Abuse Treatment Studies consortium of nine research centers, examined an organizational intervention to improve the processes used in correctional settings to assess substance abusing offenders, develop case plans, transfer this information to community-based treatment agencies, and monitor the services provided by these community based treatment agencies.

**Methods/Design:**

A multi-site cluster randomized design was used to evaluate an inter-agency organizational process improvement intervention among dyads of correctional agencies and community based treatment agencies. Linked correctional and community based agencies were clustered among nine (9) research centers and randomly assigned to an early or delayed intervention condition. Participants included administrators, managers, and line staff from the participating agencies; some participants served on interagency change teams while other participants performed agency tasks related to offender services. A manualized organizational intervention that includes the use of external organizational coaches was applied to create and support interagency change teams that proceeded through a four-step process over a planned intervention period of 12 months. The primary outcome of the process improvement intervention was to improve processes associated with the assessment, case planning, service referral and service provision processes within the linked organizations.

**Discussion:**

Providing substance abuse offenders with coordinated treatment and access to community-based services is critical to reducing offender recidivism. Results from this study protocol will provide new and critical information on strategies and processes that improve the assessment and case planning for such offenders as they transition between correctional and community based systems and settings. Further, this study extends current knowledge of and methods for, the study of evidence-based practice adoption and implementation.

**Electronic supplementary material:**

The online version of this article (doi:10.1186/2194-7899-2-1) contains supplementary material, which is available to authorized users.

## Background

Screening and assessment are clinical processes used to detect and then determine the extent, pervasiveness, or severity of presenting problems or issues by patients in a variety of health and other service settings. For individuals engaged in criminal justice or correctional systems, these screening and assessment processes should identify and evaluate criminogenic risks, including mental health and drug abuse problems, in order to tailor correctional supervision and rehabilitative services to those who need them (Lowenkamp and Latessa 2005; Taxman and Thanner 2006; Welsh and Zajac 2004). Several screening and assessment tools have been validated for use in both substance abuse treatment and correctional programs. These instruments assess static and dynamic individual factors that can aid in informing the intensity and course of substance abuse treatment and correctional supervision that is expressed in an offender case plan. Linking screening and assessment information to offender case plans is a cornerstone of the Risk-Need-Responsivity principle, a feature of evidence-based correctional programming (Andrews et al. 1990).

There is some evidence that the assessment and case planning processes used in criminal justice and correctional settings are less than optimal (Taxman, Cropsey et al. 2007; Taxman, Perdoni and Harrison Perdoni and Harrison 2007; Belenko and Peugh 2005). In one recent national survey, only 58% of institutional correctional agencies (prisons and jails) in the United States reported use of standardized assessment instruments, with community correctional agencies (probation and parole) showing even lower rates of utilization (Taxman, Cropsey et al. 2007). The lack of validated screening and assessment processes in correctional settings represents a significant threat to the overall effectiveness of correctional services. In the absence of effective offender assessment processes, responsive supervision and treatment plans cannot be developed, putting the offender at risk of re-offending, and the public at risk of victimization and crime.

Systematic approaches to organizational change within correctional and criminal justice settings date back to the 1960s with a focus on prison reform and community policing (Duffee et al. 1986; Toch 1969; Toch et al. 1975). Nonetheless, rigorous research related to the implementation of specific, targeted, evidence-based supervision and/or clinical practices is lacking in criminal justice systems. The supervision and clinical practices associated with the assessment, case planning, and referral to community-based substance abuse treatment of offenders is an important dimension of the criminal justice system. These practices take on particular importance when one considers the growth in the offender population in general, the prevalence of community correctional supervision, the high prevalence of substance use disorders among individuals under correctional supervision, and the resulting reliance on interagency (correctional-treatment) models of service delivery (Klofas et al. 2010).

### Aims and objectives

The aim of this study is to test an interagency implementation strategy in linked correctional and community-based treatment systems to improve the assessment and case planning processes that these agencies and their staff perform as they coordinate substance abuse treatment and services for offenders transitioning between these two systems. Since correctional and community-based treatment systems are heavily influenced by state-level policies and funding resources, a randomized cluster design, with clusters formed at the state level, controls for the effects of the exogenous policy environment. The implementation strategy consists of externally facilitated organizational coaching and interagency Local Change Teams (LCTs) that include individuals in staff and managerial positions from correctional and community treatment agencies. The objectives of this study are threefold: (1) improve the quality of the assessment and case planning processes of correctional-based agencies; (2) assess the effectiveness of an externally facilitated, interagency change team process in implementing targeted process improvements; and (3) evaluate the impacts and determinants of this change process upon staff behavior, attitudes, and quality of assessment and case planning processes.

### Significance

The social significance of this study lies in its context within criminal justice systems and its focus on the processes of offender assessment and case referral, an interagency juncture long recognized to be faulty and ill-devised (Taxman, Cropsey et al. 2007; Taxman, Perdoni and Harrison Perdoni and Harrison 2007). As society continues to grapple with the explosion in incarceration and community supervision, it is critical to identify more effective and efficient processes and procedures for correctional systems to better assess the needs of offenders and to provide this information to community-based providers to enable them to deliver evidence-based treatment to offenders.

The utilization of organizational coaching and external facilitation has been well documented in the research literature, and change teams have been a common element in implementation change processes, but the facilitation of *interagency* change teams, which involve the needs, abilities, and priorities of different systems of care, such as correctional agencies and treatment programs, has been less well studied (Aarons et al. 2009).

The research significance of this study lies in its application of an innovative research design and the utilization of multi-method measurement processes to study community-generated process improvement targets. The diversity of process improvement action targets taken on by the LCTs and the reliance upon newly developed non-psychometrically validated instrumentation creates potentially significant analytic and interpretation challenges. The lessons learned from this study may contribute to a better understanding of appropriate and efficacious methodological approaches to the study of organizational change and implementation.

## Methods/Design

### Intervention

The OPII tested the effects of an organizational implementation strategy upon improvements in an intervention strategy, consistent with the emergent field of implementation research that distinguishes intervention strategies (those activities delivered to program recipients) from implementation strategies (those activities delivered to organizations and providers delivering the intervention strategy) (Proctor et al. 2009). The intervention strategy that we targeted was the linked processes of offender assessment, case planning, and referral to community based treatment shared by correctional agencies and linked community based treatment agencies. The implementation strategy tested was an organizational intervention consisting of externally facilitated organizational coaching provided to interagency LCTs.

The implementation strategy of the OPII is similar to the NIATx model that uses a change team and coach to bring about process improvements in behavioral health settings (McCarty et al. 2007). However, the OPII differs from the NIATx model in the following ways: (1) the facilitator in OPII was more engaged with the LCT than a NIATx coach would be; (2) the OPII had defined phases with phase-specific activities and reports in contrast to the more open-ended process of NIATx; (3) the OPII did not use rapid-cycle testing, Plan-Do-Study-Act (PDSA) processes, which are core elements of NIATx; and (4) the OPII targeted *interagency* change team processes (corrections and treatment) while NIATx involves a single agency.

Each LCT was made up of individuals from a participating correctional agency and at least one community-based substance abuse treatment agency that received referrals from the correctional agency. The LCT ranged in size from 6 to 10 individuals, and included individuals with responsibility for the assessment, case planning, referral processing, and substance abuse treatment planning functions. The size and composition of LCTs depended upon the local context and the organizational characteristics of the participating correctional and provider agencies.

Each LCT had a Local Change Team Leader (LCTL). The individual designated for this position was expected to have direct line communications to the chief executive officer (e.g. commissioner, chief probation officer, parole board chair, or parole director) of the corrections partner agency in which the OPII was being conducted. The LCTL served as the communication and decision-making pipeline with the corrections agency CEO and facilitated logistical and operational change processes identified by the LCT.

The facilitator was an individual who worked with the LCT throughout the organizational improvement process. Each research center, in cooperation with the relevant correctional agency partner, selected facilitators who were under the employ of the RC. In general, facilitators had previously worked directly with agency providers in some capacity and possessed credentials and experience that provided credibility with the LCT.

Facilitators helped LCTs stay on track and on task as they engaged in a structured, five-phase model of assessing and improving the quality of their interagency assessment and case planning mechanisms within correctional and community treatment systems. The five structured phases of the OPII and the planned duration of each phase were as follows: (1) Team Development (1–2 months); (2) Needs Assessment (3–4 months); (3) Process Improvement Planning (3–4 months); (4) Implementation (6 months); and (5) Follow-Up/Sustainability (6 months).

During the Needs Assessment phase, the LCT engaged in a variety of information gathering and group decision-making techniques to critically examine and prioritize gaps or capacities in four core quality dimensions of their shared assessment and case planning processes: (1) Was the correctional agency using evidence-based and validated means for assessing the needs of offenders? (2) Were these needs identified and prioritized in the resulting case plans developed by the correctional agency? (3) Did the correctional agency share this assessment and case plan information with their referring community-based treatment providers, and did the providers find this information useful? (4) Did the community based agency provide services that addressed the needs of the offenders? The LCT, with assistance provided by the facilitator, used information gathered during the Needs Assessment to identify improvement goals, created a Process Improvement Plan (PIP), and carried out the implementation activities they had set out for themselves (see Table 1). During the Sustainability phase, the LCT developed a plan to continue implementation activities and the planned withdrawal of the facilitator. During this phase, attention was paid to determining if continuing work on the PIP goals was needed, if new goals were needed, and whether the LCT process would continue.

**Table 1:** **Core dimensions of the assessment continuum**

**Measurement and instrumentation**	This dimension is concerned with the breadth and quality of instruments that a correctional agency uses to identify the strengths, weaknesses, and service needs of substance-using offenders. Nine domains have been identified as being fundamental to a high quality assessment of offenders with substance use disorders:
	1. History and patterns of substance abuse
	2. History of and engagement in drug treatment
	3. Motivation for treatment
	4. History of mental illness
	5. Suitability for pharmacological treatment
	6. Medical history
	7. HIV/AIDS status and risk factors
	8. Criminal behavior
	9. Criminogenic risk factors
	In addition to focusing on the comprehensiveness of the assessment, this dimension is also concerned with the psychometric properties of the instruments.
**Integration with the case plan**	This dimension is concerned with the extent to which the correctional case plan explicitly addresses each of the nine assessment domains. It also seeks to gauge efficacy and suitability to the needs of the offender as called for in the written problem statement, goals, objectives, and suggested interventions.
**Conveyance and utility**	This dimension is concerned with the extent to which community-based treatment programs receive the information contained in the corrections agency case plan and with the degree to which the programs find the information useful in arranging services for clients.
**Service activation/provision**	This dimension is concerned with whether the client is engaged in community treatment, with the type and nature of services received, and with communication between agencies about the treatment.

Cross-site fidelity among those individuals serving as facilitators was attained through three mechanisms. First, a facilitators’ manual^a^ was developed prior to the launch of the study. Second, weekly learning circle calls among the facilitators, which included discussion of site updates and group problem-solving of organizational impediments and challenges, assisted in enhancing cohesion and consistency in approach. Third, a secure web-portal utilized by the facilitators to report the frequency, duration, and type of contacts that they had with members of their change teams, along with descriptive progress notes, allowed the research team to monitor the activities of the facilitators.

Nine research centers (RC) participated in this study, with each RC comprised of correctional/criminal justice (CJ) agencies, community-based treatment agencies, and researchers. The role of each RC in the OPII study was to create and participate in the implementation strategy and to participate in workgroups that addressed issues such as implementation, data collection and quality, analysis, and publication. Resources and incentives provided by the research centers to the CJ and community providers varied across centers, but included opportunity and nominal funding for education (continuing education units), improvements in delivery of services, and development of an implementation process that could be used after the research was completed.

### Research design

**Figure 1:**
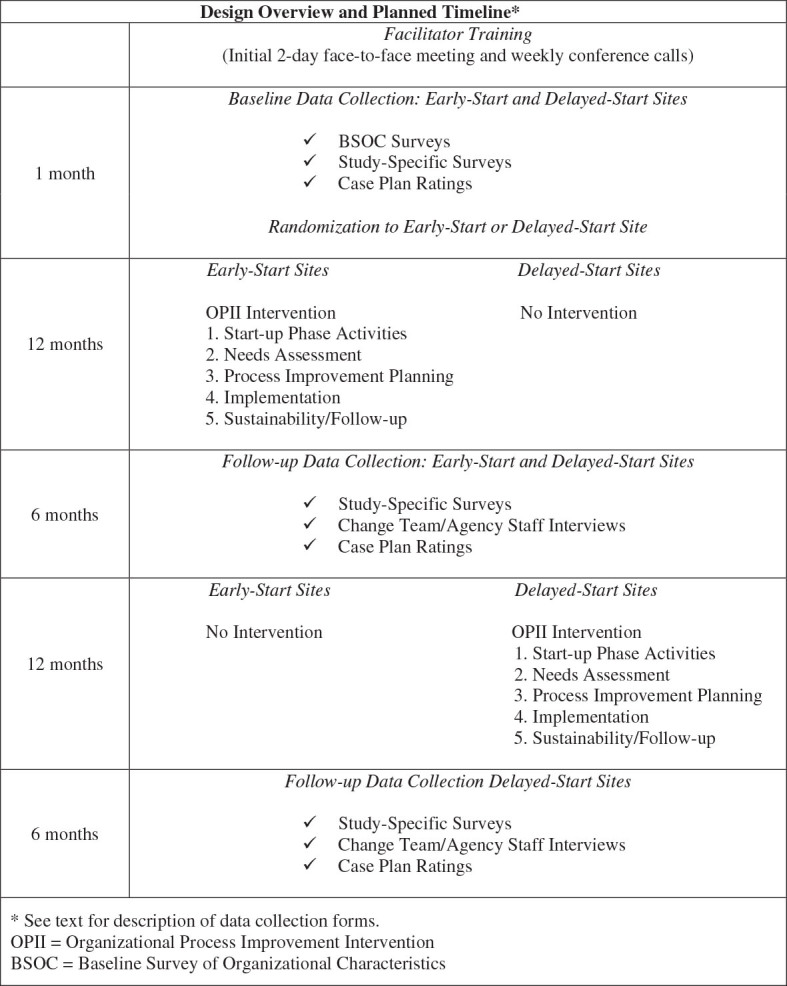
Design overview and planned timeline*.

Evaluation of the OPII used a multi-site cluster randomized design. Organizational clusters consisted of linked correctional and one or more community-based substance abuse treatment agencies providing correctional and substance abuse treatment services to common clients. Nine research centers contributed at least two clusters both of which were located within the same state. In this design, one cluster was randomly assigned to an early start condition, and the other cluster was assigned to a delayed start condition (see Figure 1). Randomization assignment was conducted by the cross center research workgroup using the randomization function in Excel for each research center prior to their kick-off meeting. Early-Start sites began the OPII, while the Delayed-Start sites maintained business as usual without any additional intervention. The Delayed-Start LCT was supposed to begin the OPII after approximately 12 months, or when the Early-Start site LCT had completed the Implementation phase of the OPII. The time required for each LCT to complete each phase of the OPII varied, based upon the existing cohesion among the LCT members, local contextual factors, and the complexity of the system and resulting goals set by the LCT. Within each research center, the Early and Delayed-Start sites were chosen so their systems of care were relatively independent.

Cluster randomization designs are more complex than randomization at the individual level, in part because intra-cluster inter-correlations (e.g., individual-level factors) introduce a design effect that must be estimated for sample size determinations and incorporated into analyses of study data (Glynn et al. 2007). However, cluster randomized designs are well suited to studies in which the intervention is targeted at the organizational rather than at the client level, as was the case for the OPII. Initially, the Delayed-Start sites served as the comparison for the Early-Start sites in that they continued to conduct their assessment, case planning, and referral procedures as usual. Since these procedures varied considerably across the correctional systems involved in the study, there was no uniform comparison condition across all sites.

### Sample

The CJDATS research centers each recruited two correctional agencies, and each correctional agency had one or more community treatment providers. Correctional settings included prisons, probation and parole units. Most (19) of the participating correctional settings served adults, but two of them served juveniles. There are 10 sites (clusters) in each study condition, for a total of 21 study sites.^b^ As indicated earlier, staff members participating in the LCT included representatives from both correctional and community-based treatment agencies who conducted assessments and/or prepared case plans and those who held management or clinical supervision positions. Each LCT included 6–10 staff members. Thus, the total number of LCT members ranged from about 120 to 240. Although the LCT members were the main participants in the study, correctional and treatment staff who were not members of the LCT were included in the administration of some of the surveys.

### Data collection and measures

#### Outcomes

The outcomes of primary interest were those related to change in the intervention being provided to offenders, namely, assessment and case planning processes. Measures of intervention outcomes included congruence between assessed needs and case plan recommendations, quality of the content of the case plan, conveyance of case plans to community treatment providers, and cross-organizational coordination.

Also of interest were the implementation outcomes of the facilitated change intervention process itself, which are related to fidelity and acceptability of the activities of the OPII. It was hypothesized that success in achieving process improvement goals, with regard to the assessment and case planning process, were dependent upon commitment to the intervention by members of the LCT, satisfaction with the facilitation, executive management support, interagency collaboration, and the quality and intensity of facilitation.

#### Quantitative data collection

Quantitative data collected for this study included structured ratings of correctional agency offender case plans and surveys of members of the LCT and other organizational staff in the participating agencies.

##### Case plan ratings

The Assessment and Recommendations for Treatment Rating Form (ART/RF) provided ratings of four quality dimensions of the case plans. These dimensions included: (1) Measurement (the problem or service needs assessed by a given agency); (2) Integration with the Case Plan (the degree to which the case plan targets needs identified); (3) Conveyance (evidence that the case plan was shared with the community based treatment provider); and (4) Services Activation (evidence that the community-based treatment provider delivered services in accordance with the needs identified in the case plan). Case plan ratings were collected before the start of the intervention (baseline), during the intervention (Needs Assessment phase, Process Improvement Plan phase, Implementation phase), and during the Sustainability/Follow-up phase. In each period, research staff randomly selected five case plans per month from agency records and rated them using the ART-RF. Case plans from the Delayed-Start sites were rated during the same period of time as for the Early-Start sites. Composite scores for each of the four quality dimensions were calculated for the five cases sampled each month, generating four ratings (Measurement, Integration, Conveyance, and Services Activation) per month.

##### BSOC Scales

The Baseline Survey of Organizational Characteristics (BSOC) describes the organizational characteristics, climate, and culture of the participating sites across the three CJDATS studies (the OPII study, as described here, the MATICCE study, which was designed to improve access to medication-assisted treatment, and the HIV-STIC study, which was intended to improve the HIV continuum of care). The BSOC was adapted from previously developed and validated instrumentation, including the TCU Survey of Organizational Functioning (TCU-SOF) (Lehman et al. 2002). There are different versions of the BSOC, with item wording tailored to the type of respondent: treatment staff, correctional staff, treatment director, and correctional director. In addition, treatment executive and correctional executive versions of the BSOC collected data on number of staff, staff turnover, types of services provided, admissions, caseload, and budget.

##### Other surveys

Other survey instruments (listed in Table 2) provide information on staff perceptions of the assessment-case planning process, conveyance and use of assessments and case plans by community treatment agencies, goal commitment by members of the LCT, working alliance between the facilitator and members of the LCT, completion of implementation tasks per phase, perceived management support for the process improvement process, and staff satisfaction with the OPII. To assess costs of the intervention, members of the LCT were asked to report monthly on the number of hours they spent on LCT activities.

**Table 2:** **OPII Variables, instruments, and assessment schedule**

Construct/Variable	Instrument	Who assessed	When assessed
Organizational Climate and Culture	Baseline Survey of Organizational Characteristics	Change Team	Baseline
		Correctional Staff	
		Treatment Staff	
		Correctional Managers	
		Treatment Managers	
Quality of Assessment and Case Planning	Assessment and Recommendations For Treatment Rating Form	Correctional facility case plans	Monthly sample of case plans from baseline through end of follow-up
Goal Commitment	Goal Commitment	Change Team	Baseline
			End of Planning Phase
Management Support	Management Support (Change Team; Management Versions)	Change Team	Baseline
		Change Team Supervisors	End of Planning Phase
			End of Implementation Phase
Perceptions of Assessment Process	Staff Perceptions of Assessment Process	Change Team	Baseline
		Correctional and Treatment involved in assessment and treatment planning	End of Implementation Phase
			End of Follow-up Phase
Use of Case Plans	Community Provider Assessment of Conveyance and Use of Case Plans	Community Treatment Provider Administrator	Baseline
			End of Implementation Phase
			End of Follow-up Phase
Satisfaction	Staff Satisfaction (Change Team; Management Versions)	Change Team	End of Planning Phase
		Change Team Supervisors	End of Implementation Phase
Working Alliance	Working Alliance (Change Team; Facilitator Versions)	Change Team	End of Needs Assessment Phase
		Facilitator	End of Implementation Phase
Interagency Collaboration	Services Coordination Scale (from BSOC)	Change Team	Baseline
		Correctional Staff	End of Follow-up Phase
		Treatment Staff	
		Correctional Managers	
		Treatment Managers	
Cost	Change Team Time Report	Change Team	Every month during the intervention
Implementation	Implementation Checklist	Research Staff	Monthly
Attitudes toward and Experiences with Implementation Strategy	Qualitative Interviews	Change Team	End of Planning Phase
		Facilitators	End of Implementation Phase
		Correctional Staff	End of Follow-up Phase
		Treatment Staff	

#### Qualitative data collection

Semi-structured interviews with members of the LCTs and other staff (front-line staff, and administrators) of the participating agencies were conducted periodically throughout the OPII intervention, specifically at the end of the Process Improvement Planning Phases, the end of the Implementation Phase and at the end of the Sustainability/Follow-Up Phase. Respondent interviews were valuable to understand and clarify the experiences, motivations, and underlying attitudes of participants involved in change projects (Tracy 2013). The interviews, which were conducted across all research sites, followed standardized interview guides for each phase of the project focused, although interviewers were encouraged to use a “conversational give-and-take” style (Lindlof and Taylor 2002) to probe for additional detail and ask clarifying questions when necessary. Interview guides focused on the respondents’ experiences with and perspectives regarding the implementation of the OPII, both from an insider (LCT member) and outsider (line staff, administrator) perspectives, and asked respondents to report about important issues including, for example: LCT cohesion and group process, specific goals and their feasibility, personal and team participation, facilitator strengths/weaknesses, effects of the change process, etc. These interviews were particularly useful for identifying unanticipated factors that affected the success of the change process.

Interviews were conducted by members of each research center, either in person or over the phone. Interviews were audio recorded, transcribed, fact-checked, stripped of identifying information, and then analyzed using a multi-part group and individual coding process.

### Aims and/or hypotheses

#### Hypotheses

The primary hypotheses of the study are that enhancements or improvements in each of the following outcomes occur only after the introduction of a specific and structured process improvement initiative (OPII):

1. The level of congruence between transitional offender assessments and case plans.
2. The level of presence of accepted principles of case plan development in case plans.
3. The percentage of case plans forwarded from correctional agencies to community treatment programs.
4. The level of the use of case plans by community-based substance abuse treatment programs.
5. Staff perceptions of the assessment-case planning process.

Secondary hypotheses are concerned with factors affecting the degree of success that LCTs experienced in achieving the goals they established for themselves. LCTs’ success in achieving the goals for their Process Improvement Plans (PIP) were expected to be positively related to:

1. The degree to which LCTs exhibit fidelity to the designated elements of OPII.
2. The degree of commitment by LCT members to achieving the goals of the plan.
3. The level of staff satisfaction with the implementation strategy.
4. The degree of management support within the organization for the intervention.
5. The strength of the working alliance between the facilitator and the LCT.

#### Implementation questions

The implementation questions for the OPII study include: (1) How are implementation outcomes related to variations across states in system characteristics, configurations of LCTs, assessment processes, and study implementation? (2) Were the improvements in assessment and case planning procedures identified by each LCT implemented as intended? (3) What does the OPII cost in terms of staff time devoted to designing and implementing the PIP? (4) Are OPII-initiated changes in assessment and case planning sustained following the end of the intervention? (5) In what ways does collaboration between organizations involved in the OPII change over the course of the intervention?

### Human subject protections

Each research center obtained Institutional Review Board approval through an established FWA-recognized entity. In most instances, approvals were also secured from participating correctional and/or treatment agency research/IRB committees. Informed consent was obtained by research participants, including staff and managers of participating agencies, at varying points of time throughout the study, depending upon data collection requirements. Baseline structured staff surveys and corresponding participant consent were administered at the time of the kick off meeting of the early start site for participants of both the Early-Start and Delayed-Start sites. Qualitative interviews occurred after the randomization. ART-RF case ratings samples began six months prior to randomization; since the ratings did not collect personal identifying information, but rather agency documentation patterns, offender consent was not required.

## Discussion

The organizational intervention under study in this paper will extend the use of interagency LCTs and externally facilitated organizational coaching to enhance the shared processes of assessment, case planning, service referral, and treatment provision processes between correctional agencies and community based treatment agencies. This study will generate and extend knowledge related to the science of implementation and organizational change in at least four key areas.

First, the study will provide some of the first evidence of the effectiveness of change teams and facilitated coaching strategies to bring about changes in organizational processes (specifically assessment and case planning) within correctional systems. While the utilization of organizational coaching and facilitation has been recognized as an effective organizational change process in correctional systems (National Institute of Corrections 2001), scant empirical evidence exists of its impact in promoting adoption and implementation of evidence-supported practice.

Second, the application of organizational change strategies such as change teams, and process improvement initiatives, such as NIATx, typically target change processes within a single organization. This study targets organizational processes within and between systems and agencies; criminal justice/correctional agencies and private, mostly non-profit community-based treatment agencies. The interagency contexts of this study, coupled with the divergence in organizational culture between correctional and treatment settings, provide unique context within which to study the complexities of bringing about enhancements in the delivery of evidence-supported client level interventions.

Third, this study provides a highly structured and rigorous approach to ensuring and documenting the fidelity of the facilitated intervention, including the development of a facilitation intervention manual and learning circles among the facilitators. These enhancements introduce significant opportunities to better understand the nature and quality of effective organizational facilitation.

Fourth, this study extends methodological approaches to the inquiry of implementation and organizational improvement in a number of ways. As noted, the use of non-validated instrumentation, most notably in the case file review process (ART-RF), but also nearly all of the survey measures, present major risks and challenges to the analysis and interpretation processes. Nonetheless, the focused efforts at construct triangulation, drawing upon multi-methods data collection (survey, chart abstraction, qualitative interviews) provide the potential for advancing measurement sophistication in this nascent field of inquiry. The reflective nature of our intervention design, one in which the speed at which the LCTs progress through the planned phases of the intervention, as well as the targeting of the process improvement goals selected by each LCT, present significant risks and challenges to analysis and interpretation. Finally, given the local setting context within which these LCTs are formed, the potential for spillover or generalization effects between early start and local start sites is an area for concern. For each of these methodological risks and liabilities, we have taken efforts to anticipate and guard against the most egregious risks, and we hope, in the process, to make significant contribution to the study of organizational improvement and implementation in general and within the unique context of correctional settings in particular.

## Endnotes

^a^Copies of the Facilitator Manual can be obtained by contacting the corresponding author.

^b^Although there were nine (9) CJDATS Researcher Centers, one Center had two sets of study sites in two states, while another Center had a total of three study sites. The remaining seven research centers fielded one cluster each, with two study sites per cluster (n = 14).

## Authors’ information

The CJDATS Assessment Workgroup members who participated in the development of the Assessment protocol are (in alphabetical order within each Research Center): David Duffee, Cassia Spohn (Arizona State University), Karen McKendrick, (National Development and Research Institutes, Inc.), Matthew Hiller, Roger Peters, Ralph B. Taylor, Gary Zajac (Temple University), Wayne Lehman (Texas Christian University), Linda K. Frisman, Colleen Gallagher, Eleni Rodis (University of Connecticut), Steven S. Martin, Cynthia Robbins (University of Delaware), Jamieson Duvall, Erin McNees Winston, Michele Staton Tindall (University of Kentucky), Bennett W. Fletcher (Bethesda, MD).

## Authors’ original submitted files for images

Below are the links to the authors’ original submitted files for images. Authors’ original file for figure 1
