# Supplementary material for: A cluster randomized trial of an organizational process improvement intervention for improving the assessment and case planning of offenders: a Study Protocol
Source: Health Justice. 2014 Jan 8;2:1. doi: 10.1186/2194-7899-2-1 (PMC4279850; doi:10.1186/2194-7899-2-1)
Supplement: Supplementary file 1 — Authors’ original file for figure 1 [file 40352_2013_6_MOESM1_ESM.pdf]

<table>
<tr><th colspan="3">Design Overview and Planned Timeline*</th></tr>
<tr><td></td><td colspan="2">Facilitator Training
(Initial 2-day face-to-face meeting and weekly conference calls)</td></tr>
<tr><td>1 month</td><td colspan="2">Baseline Data Collection: Early-Start and Delayed-Start Sites
✓ BSOC Surveys
✓ Study-Specific Surveys
✓ Case Plan Ratings
Randomization to Early-Start or Delayed-Start Site</td></tr>
<tr><td>12 months</td><td>Early-Start Sites
OPII Intervention
1. Start-up Phase Activities
2. Needs Assessment
3. Process Improvement Planning
4. Implementation
5. Sustainability/Follow-up</td><td>Delayed-Start Sites
No Intervention</td></tr>
<tr><td>6 months</td><td colspan="2">Follow-up Data Collection: Early-Start and Delayed-Start Sites
✓ Study-Specific Surveys
✓ Change Team/Agency Staff Interviews
✓ Case Plan Ratings</td></tr>
<tr><td>12 months</td><td>Early-Start Sites
No Intervention</td><td>Delayed-Start Sites
OPII Intervention
1. Start-up Phase Activities
2. Needs Assessment
3. Process Improvement Planning
4. Implementation
5. Sustainability/Follow-up</td></tr>
<tr><td>6 months</td><td colspan="2">Follow-up Data Collection Delayed-Start Sites
✓ Study-Specific Surveys
✓ Change Team/Agency Staff Interviews
✓ Case Plan Ratings</td></tr>
<tr><td colspan="3">* See text for description of data collection forms.
OPII = Organizational Process Improvement Intervention
BSOC = Baseline Survey of Organizational Characteristics</td></tr>
</table>
